# Moderate coffee and tea consumption is associated with slower cognitive decline: data from UK Biobank

**DOI:** 10.1002/alz.094923

**Published:** 2025-01-09

**Authors:** Samantha L Gardener, Kelsey R Sewell, Belinda M. Brown, Ralph N Martins, Stephanie R Rainey‐Smith

**Affiliations:** ^1^ Edith Cowan University, Perth, Western Australia Australia; ^2^ Alzheimer’s Research Australia, Perth, Western Australia Australia; ^3^ AdventHealth Research Institute, Neuroscience, Orlando, FL USA; ^4^ Murdoch University, Perth, Western Australia Australia; ^5^ Department of Biomedical Sciences, Macquarie University, Macquarie Park, NSW Australia; ^6^ Alzheimer's Research Australia, Perth, Western Australia Australia; ^7^ School of Psychological Science, University of Western Australia, Crawley, Western Australia Australia

## Abstract

**Background:**

Worldwide, coffee and tea are two of the most popular beverages consumed. Studies have suggested a protective role of coffee and tea, including reduced risk of Alzheimer’s disease. However, longitudinal data from large cohorts of older adults reporting associations of coffee and tea intake with cognitive decline is limited.

**Method:**

Cognitively unimpaired participants (n = 8,451) from UK BioBank (≥60 years of age) with at least 2 follow up visits (average follow up time 8.83 years) were included. Baseline self‐report coffee and tea intake (cups per day) were collected using a questionnaire. Cognitive assessment included Pairs Matching test (errors), Reaction Time test, Numeric Memory test, and Fluid Intelligence. Linear mixed models assessed the relationship between categorical coffee and tea intake and cognitive outcomes, including covariates age, sex, qualification, Townsend deprivation index, ethnicity, Apolipoprotein E ε4 status, and body mass index.

**Result:**

Daily coffee intake predicted the slope of decline in Fluid Intelligence across follow‐up (*β* = ‐0.02, *SE* = 0.007, FDR corrected *p* = .004). Those never consuming coffee and those with moderate coffee consumption (1‐3 cups) had slower declines in Fluid Intelligence compared to those with high coffee consumption (³4 cups, *β* = 0.06, *SE* = 0.02, *p* = .005; *β* = 0.07, *SE* = 0.02, *p*<.001, respectively).

Daily tea intake predicted decline in Fluid Intelligence across follow‐up (*β* = 0.02, *SE* = 0.007, FDR corrected *p* = .044), such that those who never drank tea showed a greater decline in Fluid Intelligence compared to those who had moderate and high tea consumption (*β* = 0.06, *SE* = 0.02, *p* = .009; *β* = 0.06, *SE* = 0.02, *p* = .003, respectively).

**Conclusion:**

Our results support the hypothesis that coffee and tea intake may be a protective factor against cognitive decline, particularly for maintaining Fluid Intelligence. However, our results show an upper limit to coffee intake with a maximum of 3 cups/day being consumed for beneficial effects, and no coffee intake being more beneficial than ³4 cups. Conversely, high tea consumption provided positive effects on cognition in addition to moderate consumption, with high tea consumption being more beneficial than no tea consumption. Additional longitudinal observational and intervention studies are required to validate our findings and confirm this hypothesis.

This research has been conducted using the UK Biobank Resource under Application Number 108907.

